# The exonuclease activity of DNA polymerase γ is required for ligation during mitochondrial DNA replication

**DOI:** 10.1038/ncomms8303

**Published:** 2015-06-22

**Authors:** Bertil Macao, Jay P. Uhler, Triinu Siibak, Xuefeng Zhu, Yonghong Shi, Wenwen Sheng, Monica Olsson, James B. Stewart, Claes M. Gustafsson, Maria Falkenberg

**Affiliations:** 1 Department of Medical Biochemistry and Cell Biology, University of Gothenburg, Medicinaregatan 9 A, P.O. Box 440, SE-40530 Gothenburg, Sweden; 2 Department of Mitochondrial Biology, Max Planck Institute for Biology of Ageing, D-50931 Cologne, Germany

## Abstract

Mitochondrial DNA (mtDNA) polymerase γ (POLγ) harbours a 3′–5′ exonuclease proofreading activity. Here we demonstrate that this activity is required for the creation of ligatable ends during mtDNA replication. Exonuclease-deficient POLγ fails to pause on reaching a downstream 5′-end. Instead, the enzyme continues to polymerize into double-stranded DNA, creating an unligatable 5′-flap. Disease-associated mutations can both increase and decrease exonuclease activity and consequently impair DNA ligation. In mice, inactivation of the exonuclease activity causes an increase in mtDNA mutations and premature ageing phenotypes. These mutator mice also contain high levels of truncated, linear fragments of mtDNA. We demonstrate that the formation of these fragments is due to impaired ligation, causing nicks near the origin of heavy-strand DNA replication. In the subsequent round of replication, the nicks lead to double-strand breaks and linear fragment formation.

## Abstract (web summary)

Mitochondrial DNA (mtDNA) polymerase γ has a 3′–5′ exonuclease proofreading activity. Here, the authors show it is required for creating ligatable ends during mtDNA replication, and inactivation of the activity in mice causes strand-specific nicks in DNA and the formation of linear mtDNA fragments.

## Graphical abstract

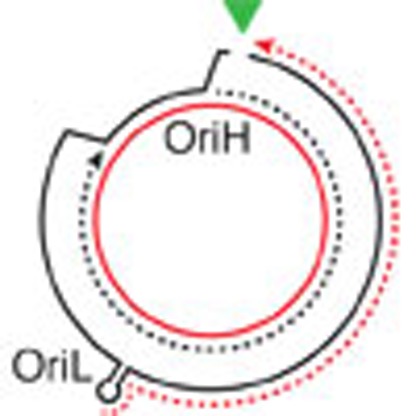

Mammalian mitochondrial DNA (mtDNA) is replicated by DNA polymerase γ (POLγ), a heterotrimeric complex consisting of a catalytic POLγA subunit and two accessory POLγB subunits. The POLγA subunit contains a 3′–5′ exonuclease domain that is connected to the polymerase domain via a spacer region^1^. The exonuclease activity corrects mistakes during mtDNA replication by reversing the direction of the polymerase and excising incorrectly introduced nucleotides. Mutations in the exonuclease domain can cause mtDNA depletion and deletions, which in turn impair ATP production and result in human diseases, including progressive external ophthalmoplegia and Alpers syndrome^2^. Accumulation of damaged mtDNA, including deletions, is also associated with normal ageing in humans and with age-related pathologies such as neurodegenerative disease^3^,^4^,^5^,^6^,^7^.

According to the strand-displacement model for mtDNA replication, DNA synthesis is continuous on both strands^8^,^9^. To ensure proper coordination of DNA synthesis, the mitochondrial genome contains two specific origins of DNA replication, one for each DNA strand: the heavy-strand origin (OriH) and the light-strand origin (OriL). After initiation at OriH, leading-strand DNA synthesis displaces the parental heavy strand (H-strand), which is covered by the mitochondrial single-stranded DNA-binding protein (mtSSB)^10^. When the replication machinery has synthesized two-thirds of the leading-strand DNA it reaches OriL, which becomes activated and serves as an initiation site for L-strand DNA synthesis in the opposite direction^8^,^9^,^11^. The strand-displacement model also suggests that termination of DNA replication, an event often overlooked, will take place near OriH on one of the two daughter molecules and at OriL on the other^8^. The final step of mtDNA replication will require ligation of nicked DNA to produce continuous circular double-stranded DNA (dsDNA). In mammalian mitochondria, the responsible enzyme is DNA ligase III (Lig3), which functions in both DNA repair and replication^12^. Loss of Lig3 leads to mtDNA depletion and embryonic lethality in the mouse^13^.

When the nuclear DNA polymerase-δ encounters the 5′-end of a previous Okazaki fragment on the lagging strand, or at the termination point of DNA replication, it undergoes a process known as idling, which entails successive cycles of polymerization and 3′–5′exonuclease degradation at the nick, and is important for efficient ligation^14^. POLγ has also been shown to idle at nicks, but the requirement of this function for proper ligation has not been investigated^15^. *In vivo*, the ligation reaction may also be aided by various nucleases, including DNA2 and FEN1, which can remove 5′-flaps formed during the idling process or longer flaps created by excessive strand displacement^16^,^17^,^18^,^19^,^20^.

Wild-type (WT) POLγ is unable to replicate dsDNA without the aid of the TWINKLE DNA helicase^21^. In contrast, an exonuclease-deficient version of POLγ (EXO-) can use dsDNA as a template and synthesize short stretches of DNA even in the absence of a DNA helicase, a process called strand-displacement DNA synthesis^15^,^22^. This effect is not specific to exonuclease-deficient POLγ, as inactivation of exonuclease activity also causes strand-displacement DNA synthesis with other DNA polymerases^23^,^24^,^25^,^26^,^27^,^28^.

The exonuclease activity of POLγ is also required for proper mtDNA replication *in vivo*. Mice in which EXO- has replaced WT POLγ (mutator mice) have increased levels of mtDNA point mutations and contain very high levels of truncated, linear mtDNA fragments^29^. Interestingly, the mutator mice also develop premature ageing phenotypes, supporting a causative role for mtDNA mutations in mammalian ageing. How the linear mtDNA fragments arise is still not understood, but they comprise the entire major arc between the replication origins, OriH and OriL^29^,^30^.

Here we demonstrate that the exonuclease activity of POLγ is required for efficient DNA ligation. If the exonuclease activity is abolished, POLγ fails to halt at 5′-ends and instead continues DNA synthesis into duplex DNA regions. This strand-displacement activity leads to the creation of a 5′-flap that is an inefficient substrate for ligation. Furthermore, we identify a number of disease-causing mutations that increase or decrease the exonuclease activity of human POLγ and as a consequence impair normal mtDNA ligation. We also follow up our findings with *in-vivo* analysis of replication intermediates in the mutator mice. DNA ligation in these mice is compromised, with increased levels of nicks in mtDNA that in turn may relax the superhelicity of the genome. Based on our observations, we suggest that these unligated nicks lead to double-strand breaks during the next round of mtDNA replication, causing the formation of the linear mtDNA fragments observed in mutator mice.

## Results

### Ligatable nick formation requires POLγA exonuclease activity

**Figure 1:**
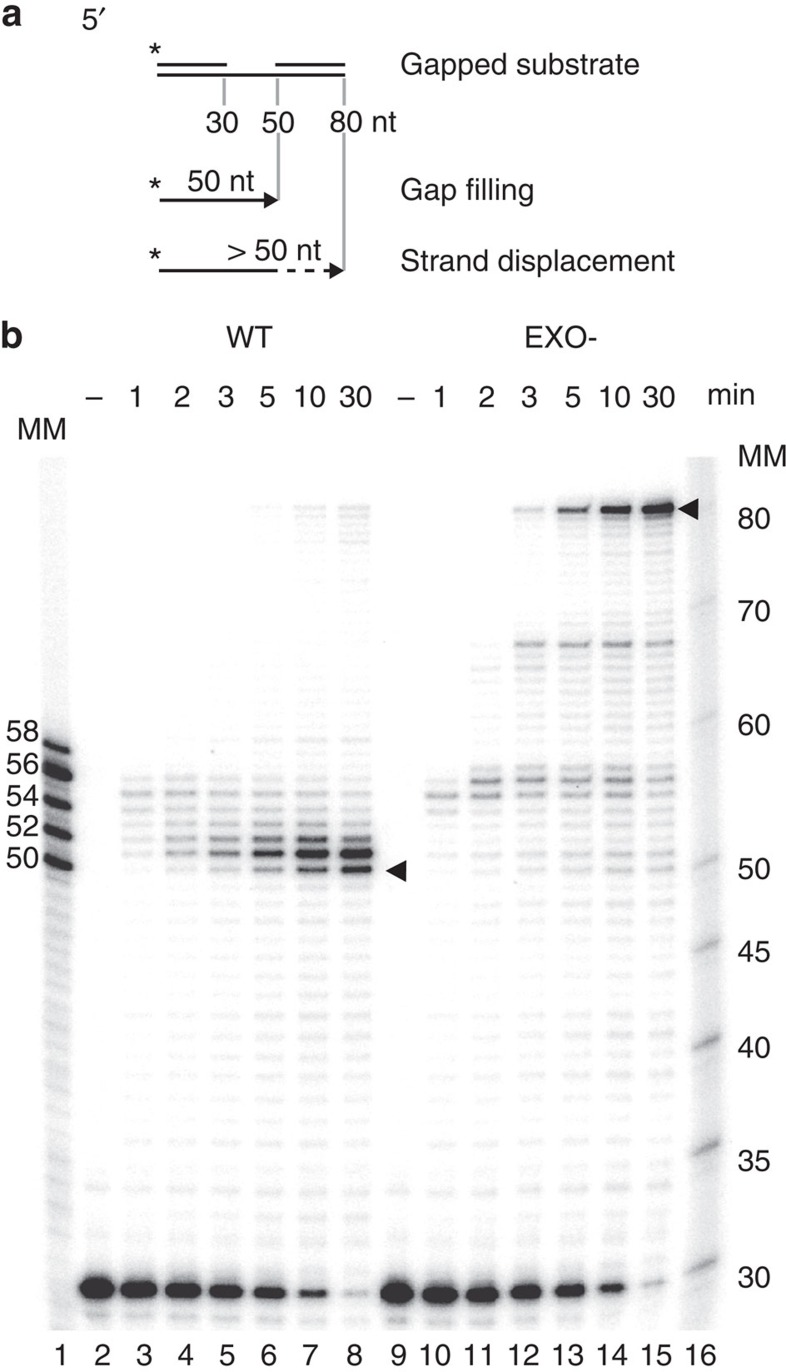
POLγ EXO- displays increased strand-displacement activity. (**a**) Diagram of the linear gapped substrate used in strand-displacement assays, with products shown below. (**b**) Strand displacement by WT and EXO- POLγ over time. WT polymerase performs limited strand displacement, up to 6 nts within the downstream duplex region, before backing up towards the nick position (arrowhead at 50 nts). EXO- polymerase rapidly displaces the downstream oligonucleotide completely (arrowhead at 81 nts). Reactions were started by the addition of POLγ. Time points are indicated above in minutes (− represents reactions where no POLγ was added). MM, molecular marker.

The exonuclease activity of POLγ can be inactivated by a single amino acid substitution in the second exonuclease motif in the POLγA subunit (D274A, hereafter referred to as ‘EXO-')^29^. The EXO- mutation causes a strand-displacement activity, that is, polymerase continues to displace downstream DNA encountered during DNA synthesis^15^,^22^. We speculated that failure to stall at the 5′-end of downstream DNA would lead to the formation of a 5′-flap, which is incompatible with ligation. To address this possibility, we used a linear substrate with a 20-nt long single-stranded gap and investigated how actively synthesizing WT and EXO- POLγ behave when encountering a free 5′-DNA end. The template used was constructed from an 80-nt-long oligonucleotide annealed to a 30-nt radioactively labelled upstream primer and a 30-nt downstream blocking oligonucleotide (Fig. 1a). Precise gap filling and creation of ligatable ends will extend the primer to 50 nts. At early time points (1–3 min), WT POLγ did not halt at the 5′-end of the downstream blocking primer, but extended up to 6 nts into the duplex region (Fig. 1b). After 5 min, bands at positions 50–52 became the dominant species, suggesting that POLγ had backed up towards the nick. EXO- POLγ behaved similarly to WT at the earliest time point, but later the enzyme continued to polymerize into the double-stranded region, eventually completely displacing the downstream strand to produce a main product of 81 nts in length by 5 min (Fig. 1b, lanes 13–15). Please note that the extra nucleotide (the template is only 80 nts long) results from the terminal transferase activity of EXO- POLγ (shown in Supplementary Fig. 1).

To further analyse strand displacement, we used the same substrate, but now labelled the downstream oligonucleotide (Supplementary Fig. 2). Confirming the above results, we observed increased strand displacement with EXO- POLγ compared with WT POLγ at all time points. By 30 min of DNA synthesis, almost 50% of the downstream oligonucleotide was completely displaced by EXO- POLγ compared with <5% for WT POLγ. We therefore concluded that the exonuclease activity of POLγ is required to limit POLγ strand-displacement activity and to promote idling near the nicked position.

### Efficient DNA ligation requires POLγ exonuclease activity

**Figure 2:**
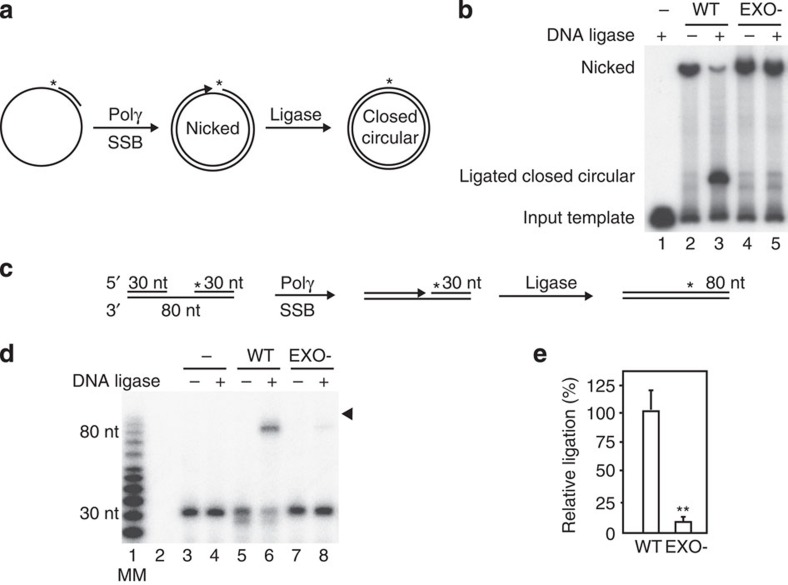
POLγ exonuclease activity is required for efficient ligation. (**a**) Diagram of the circular substrate and resulting products in replication-coupled ligation reactions. (**b**) POLγ needs functional 3′–5′exonuclease activity to produce a closed circular DNA molecule. In the coupled second strand, synthesis and ligation assay WT (lane 3) but not EXO- (lane 5) is able to form a ligated product. The presence of EtBr in the agarose gel facilitates differentiation between nicked and closed circular dsDNA molecules. (**c**) Diagram of the linear gapped substrate and resulting products in replication-coupled ligation reactions. (**d**) Ligation (80 nt product, indicated with arrow head) is severely reduced in reactions using EXO- compared with WT POLγ. Molecular marker in lane 1. (**e**) Quantification of ligation efficiency was measured as the amount of ligation product formed (bands indicated with arrowhead) against the amount of starting substrate in the absence of ligase (lane 3). WT was set to 100%. Mean values±s.e.m., *n*=3, *P*≤2.5 × 10^−3^ (Student's unpaired *t*-test).

We hypothesized that the aberrant formation of 5′-flaps by EXO- POLγ would impair ligation. To investigate this possibility, we performed coupled DNA synthesis and ligation assays using purified recombinant WT and EXO- POLγ proteins on DNA substrates. To this end, we primed an ∼3-kb circular ssDNA substrate with a 32-nt DNA oligonucleotide that had been radioactively labelled at the 5′-terminus (Fig. 2a). WT POLγ was able to polymerize until it reached the 5′-end of the primer, creating a nicked, circular dsDNA molecule (Fig. 2a, middle, and Fig. 2b, lane 2). When ligase was added, the nick was sealed (Fig. 2a, right, and Fig. 2b, lane 3). The EXO- mutant could also produce a full-circle, double-stranded product (Fig. 2b, lane 4), but did not generate ligatable ends, as no closed circular DNA molecules were observed (Fig. 2b, lane 5).

Next, we investigated coupled gap filling and DNA ligation using a substrate in which a 30-nt unlabelled upstream primer and a 30-nt radioactively labelled downstream blocking oligonucleotide were annealed to an 80-nt long template strand (Fig. 2c). Addition of WT POLγ or EXO- in the presence of mtSSB and T4 DNA ligase led to the formation of an 80-nt long ligated product (Fig. 2d, lanes 6 and 8). The ligation efficiency was however about ten times higher for the WT POLγ than for EXO- (Fig. 2e). The assay was repeated with mitochondrial Lig3 with similar results (Supplementary Fig. 3). We could therefore conclude that the exonuclease activity of POLγ is required for efficient DNA ligation *in vitro*.

### Identification of exonuclease-deficient POLγ from patients

**Figure 3:**
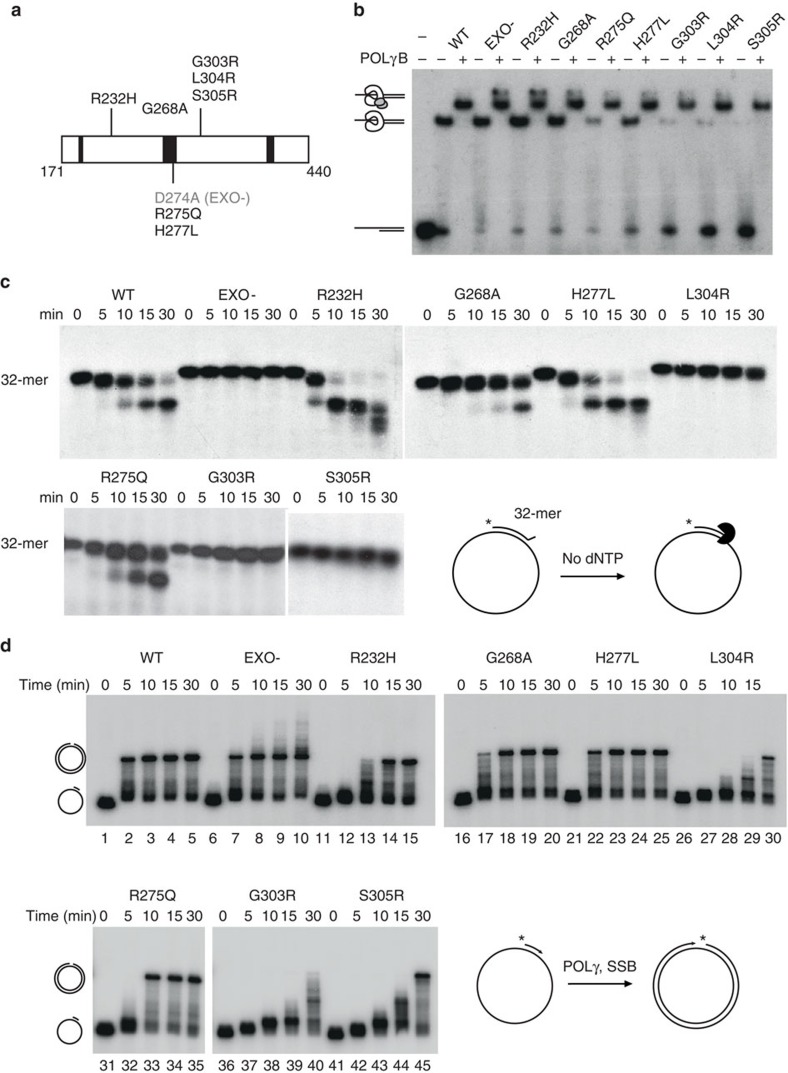
DNA exonuclease and polymerization activities of POLγ mutants. (**a**) Schematic diagram of the exonuclease domain of POL**γ**A and the position of the patient-associated mutations. The EXO- mutation D274A is indicated in grey. Black boxes represent the exonuclease motifs I, II and III. (**b**) The DNA-binding efficiencies of the POLγA mutants were tested on a primed template either as monomers or heterotrimers with POLγB (as illustrated on the left side). The R275Q, G303R, L304R and S305R mutants had markedly reduced binding activity as monomers. (**c**) Similar to EXO- POLγ, the G303R, L304R and S305R mutants have no apparent exonuclease activity. R232H has increased exonuclease activity compared with WT. The substrate is shown in the lower right quadrant. (**d**) DNA polymerization by the different POLγ proteins on a circular, 3,000-nt ssDNA template (as shown in bottom right quadrant). Synthesis rates are moderately slower for the R232H and R275Q mutants, and markedly compromised for the G303R, L304R and S305R proteins.

A number of disease-causing amino acid substitutions have been identified in the exonuclease domain of POLγ^31^ and we wanted to investigate whether these mutations also affected exonuclease activity and the formation of ligatable ends. To this end, we expressed and purified seven mutant POLγA variants in recombinant form (R232H, G268A, R275Q, H277L, G303R, L304R and S305R (Fig. 3a)). We first investigated the DNA-binding activity of the mutants in an electrophoretic mobility shift assay (EMSA), in which POLγA was incubated together with a short, radioactively labelled primed DNA template. In the absence of POLγB, the R275Q, G303R, L304R and S305R mutants all displayed reduced DNA-binding affinity compared with WT POLγ (Fig. 3b). In combination with POLγB however, all the mutants were able to bind DNA (Fig. 3b) and to synthesize a short stretch of DNA with efficiency similar to that observed for WT POLγ (Supplementary Fig. 4).

We next monitored exonuclease activity using a radioactively labelled 32-nt-long oligonucleotide annealed to pBluescript SK+ ssDNA, creating a 31-bp double-stranded region with a one-nucleotide mismatch at the 3′-end (lower right panel in Fig. 3c). The template was incubated with the WT and mutant POLγ variants for the indicated times. Of the seven proteins, G303R, L304R and S305R exhibited severe exonuclease deficiency, similar to the EXO- mutant protein, whereas the G268A and R275Q mutants had mildly impaired exonuclease activity. Interestingly, we also found a mutation, R232H, which had markedly increased exonuclease activity compared with WT. Similar results for exonuclease activity were also obtained for all mutants using a 5′-labelled primer-template substrate in the absence of dNTPs (Supplementary Fig. 4, lanes with 0 μM dNTPs).

### DNA synthesis efficiency on a longer substrate

To investigate whether our POLγ mutants could synthesize longer stretches of DNA, we used the ∼3,000-nt circular ssDNA template described above (Fig. 2a). Similar to WT POLγ, the EXO-, G268A and H277L mutants produced full-length products within 5 min (Fig. 3d, compare lanes 2, 7, 17 and 22). We observed reduced polymerization for the R232H and R275Q mutants, and even weaker activity with G303R, L304R and S305R. The L304R and S305R mutants were only able to produce full-length product after 30 min incubation (Fig. 3d, lanes 30 and 45), whereas no full-length products were observed with the G303R mutant (Fig. 3d, lane 40).

### Strand-displacement activities of POLγ mutants

**Figure 4 f4:**
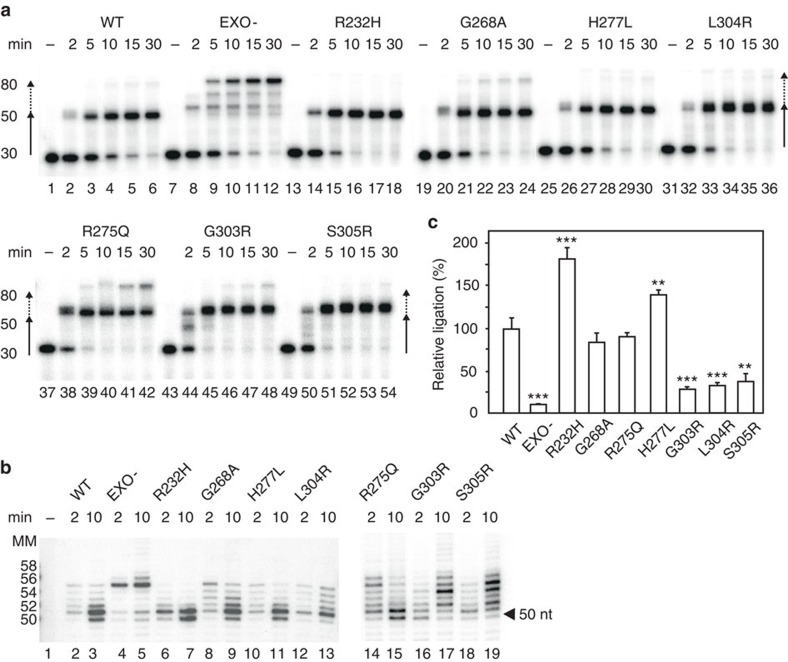
DNA strand-displacement and ligation activity of POLγ mutants. (**a**) Time course of strand displacement by purified recombinant WT and mutant POLγ proteins. The reactions were performed as depicted in Fig. 1a. Arrows: solid lines indicate gap filling; broken lines indicate strand displacement. Lanes are numbered 1–54. (**b**) Strand-displacement reactions as above resolved on a sequencing gel. The failure by G303R, L304R and S305R to reverse to the nick position at the 10 min time point is evident (arrowhead at 50 nts). The sizes of an oligonucleotide molecular marker are indicated to the left. (**c**) Quantification of ligation efficiencies (ligation product formed as percentage of substrate) of the different POLγ proteins relative to WT. The ligation assay and quantification was performed as in Fig. 2c–e. Mean values±s.e.m., WT was set to 100%, asterisks represent significant differences compared with WT (*n*=3; *P≤0.05, **P≤0.01, ***P≤0.001; one way analysis of variance).

Whereas the strand-displacement activity of EXO- polymerase was clearly visible in the long-stretch DNA synthesis assay (Fig. 3d, smear above main product in lanes 8–10), we could not see a similar effect for the disease-causing mutants. We decided to further analyse the strand-displacement activities of the different mutant polymerases over time, using the linear gapped substrate (shown in Fig. 1a). Precise gap filling will result in a band of 50 nt, whereas strand-displacement will generate products up to 80 nt. Using this template, none of the patient-related mutant proteins had strand-displacement activities as severe as the EXO- (Fig. 4a). We did however notice that three mutants, L304R, G303R and S305R, generated bands around 50 nts that appeared broader than the corresponding band generated by the WT (Fig. 4a, compare lanes 4–6 with 34–36, 46–48 and 52–54), which could be the result of entry into the duplex region and the creation of a short 5′-flap. To address this possibility, we used sequencing gels for increased resolution (Fig. 4b). In this analysis, we observed stalling at position 50–56 (that is, at the nick or 1–6 nt within the downstream dsDNA region). In keeping with its increased exonuclease activity, the R232H mutant displayed reduced strand-displacement activity, pausing at the nick (Fig. 4b, compare lanes 2–3 with 6–7). The H277L, G268A and R275Q mutants behaved similar to WT POLγ by pausing at the nick position or 1–2 nt downstream. The remaining three mutants, L304R, G303R and S305R (lanes 12-13, 16–19), stalled in a broader range within the downstream dsDNA region, creating longer 5′-flaps.

### POLγ mutations affect replication-coupled ligation

Three of the investigated mutants thus displayed increased strand displacement (L304R, G303R and S305R), whereas one displayed decreased strand displacement (R232H) compared with WT. We investigated whether these mutations also impaired ligation, as demonstrated for EXO- POLγ (Fig. 2). To this end, we performed coupled replication and ligation assays on a gapped linear substrate as illustrated in Fig. 2c,d. The experiments were performed in triplicate and the amount of ligated products was quantified. The graph in Fig. 4c compares the ligation efficiency for the different mutants relative to WT POLγ. Ligation was severely reduced with 5′-flap producing POLγ mutants (L304R, G303R and S305R). In contrast, the R232H mutant with decreased strand-displacement activity was even better than WT POLγ in producing ligatable ends.

### Increased levels of mtDNA nicks in mutator mouse cell lines

**Figure 5:**
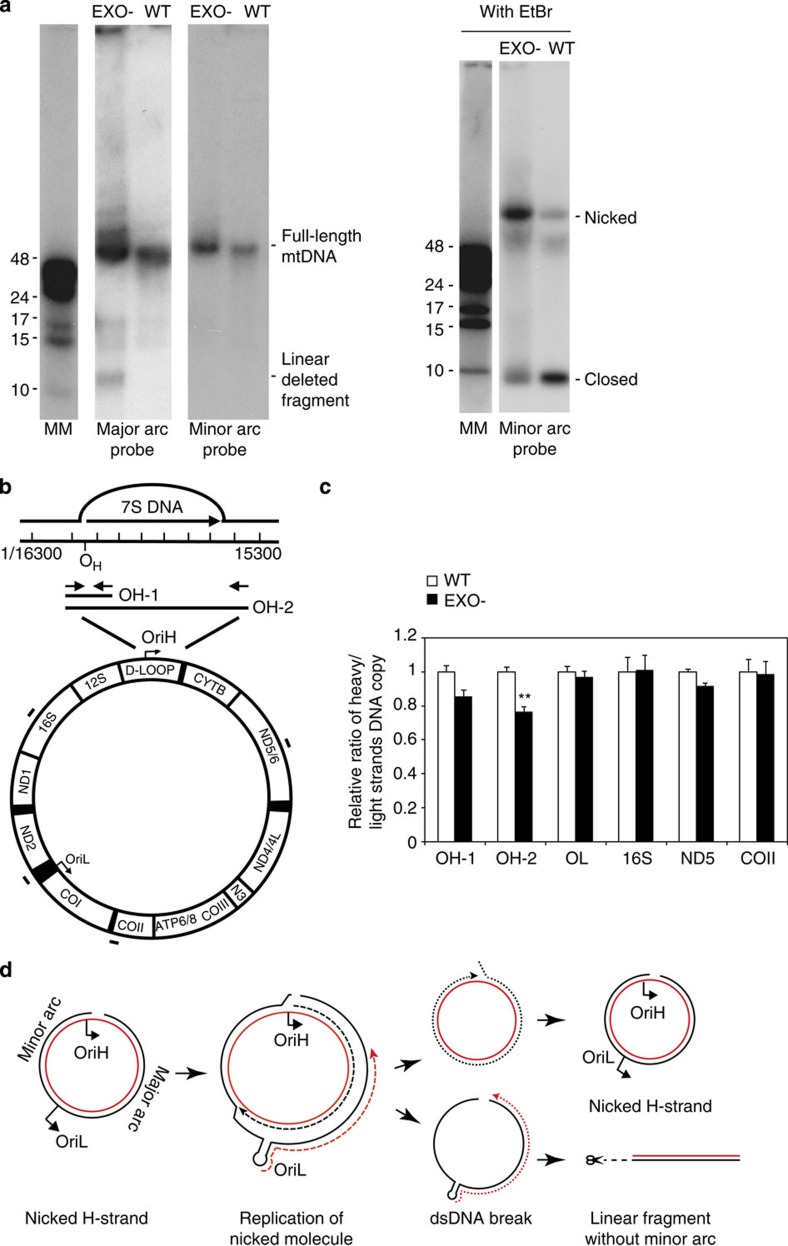
Failure to ligate can lead to nicks and linear mtDNA deletions. (**a**) MtDNA was analysed on a 0.4% agarose gel in the absence (left panel) or the presence (right panel) of EtBr. The presence of EtBr in the agarose gel facilitates differentiation between nicked and closed circular dsDNA molecules. The mtDNA was detected by Southern blotting using probes against the major or minor arc. Results were confirmed three times with two independent batches of genomic DNA preparations. (**b**) A schematic representation of the PCR template including the D-loop region. Arrows and black bars show the PCR amplification regions. (**c**) The relative ratio of DNA copies of H-/L-strands in WT and mutator (EXO-) mice. Asterisks represent significant differences compared with WT (*n*=3; *P≤0.05, Student's *t*-test). (**d**) Model of mtDNA linear deletion formation. Failure to ligate at OriH leads to a nick in the H-strand (left panel). The nick does not inhibit initiation of a new round of replication from OriH, as the intact L-strand is used as template for H-strand synthesis. DNA synthesis initiated at OriH may therefore continue full circle and create a new DNA molecule with a nick at OriH (right upper panel). In contrast, DNA synthesis initiated at OriL will result in linear, deleted fragments. In this later case, L-strand DNA synthesis will initiate from OriL and use the H-strand as template. DNA synthesis will reach the nick near OriH and a double-strand break will be formed. The molecule formed will also contain an unstable ssDNA region that will be degraded, leaving a linear double-stranded product spanning the major arc (right lower panel). Failure to ligate at OriH will thus result in the same round of replication generating two different replication products; a circular, nicked mtDNA and a linear, deleted fragment spanning OriH and OriL. Solid back line, H-strand; dashed black line, nascent H-strand; solid red line, L-strand; dashed red line, nascent L-strand.

Our studies demonstrated that mutations that change the exonuclease activity of POLγ impair ligation *in vitro*. To investigate whether these problems could also be detected *in vivo*, we used mouse embryonic fibroblasts (MEFs) derived from the mutator mouse. These homozygous knock-in mice express EXO- (D257A) in place of WT POLγ. If EXO- POLγ produces 5′-flaps and impairs ligation *in vivo*, we would expect to observe higher levels of nicked mtDNA in these animals. To address this possibility, we analysed genomic DNA isolated from WT and mutator MEFs by agarose gel electrophoresis and Southern blotting. The gel analysis was performed in the presence or the absence of ethidium bromide (EtBr) to differentiate between nicked and closed circular dsDNA molecules. In the absence of EtBr in the agarose gel, full-length mtDNA from WT and mutator cells migrated as a single band of equal size. As expected, DNA isolated from mutator cells also contained a shorter, linear DNA fragment, which was only detected with the probe against the major arc of mtDNA (Fig. 5a). In the presence of EtBr, full-length mtDNA could be separated into closed or nicked circles, which are unable to form supercoils in the presence of EtBr. Whereas a majority of mtDNA molecules isolated from WT cells were in the closed conformation, the opposite was true for mtDNA isolated from mutator cells, which mostly contained nicked circles. As controls, to verify the migration pattern of closed and nicked forms of mtDNA, the DNA was cut with different restriction enzymes (Supplementary Fig. 5). We could thus conclude that exonuclease-deficient POLγ causes the formation of nicks *in vivo*.

As initiation of mtDNA replication takes place at OriH and then proceeds unidirectionally, termination of one of the two new daughter molecules should also occur at OriH. Thus, failure to ligate after completion of DNA replication should result in mtDNA molecules that contain a nicked H-strand, but an intact L-strand near OriH. To address this possibility, we employed strand-specific PCR and monitored the levels of intact H- and L-strands in the OriH region of WT and mutator mice. Our upstream primer was located in a region not present in the linear deletion, ensuring that we were only monitoring circular mtDNA (Fig. 5b). For precise quantification, we used droplet digital PCR (ddPCR). As controls, we first quantified the levels of intact H- and L-strands in three different regions: ND5 and COXII in the major arc and 16S rRNA in the minor arc. We found equal levels of the two strands at all these locations (Fig. 5c). We next analysed a region surrounding OriH and found that the ratio between the H- and L-strand was reduced in the mutator mice as would be expected if there is a nick in the H-strand (Fig. 5c, OH-1). Owing to the displacement activity of EXO- POLγ, the nicks will be located in a broad zone rather than at a single point. In agreement with the notion, the strand bias was even stronger when we analysed a longer region, spanning the entire D-loop region (Fig. 5c, OH-2). We also analysed the OriL region and found equal levels of the two strands. This is also what is expected according to the strand-displacement replication model, as explained in the discussion (see below). Together, these results provide *in vivo* support for the notion that the exonuclease activity is required for efficient ligation and also demonstrate that the H-strand is frequently nicked in the OriH region of mutator mice.

## Discussion

MtDNA deletions underlie many mitochondrial diseases and have also been linked to biological ageing. Most naturally occurring deletions are associated with repeat sequences in mtDNA and different models have been proposed for how these arise^32^,^33^. A less-understood phenomenon is the DNA rearrangements observed in patients with MGME1 deficiency. In addition to mtDNA depletion and deletions, this mitochondrial syndrome is also associated with a linear, 11-kb mtDNA fragment that spans the entire major arc of mtDNA^34^. Interestingly, the same type of linear fragments are also seen in mutator mice, in which the exonuclease activity of POLγ has been inactivated by a single amino-acid substitution^30^. The ends of the linear fragments produced in mutator mice have been mapped by sequencing of S1-digested mtDNA products^30^. One end maps to the D-loop region and the other to the OriL region.

POLγ exonuclease activity is required for proofreading during mtDNA synthesis. In the present work, we have investigated another aspect of this activity: its importance for the formation of ligatable ends during mtDNA replication. We demonstrate that inactivation of the exonuclease activity will increase strand-displacement activity, that is, DNA synthesis will continue beyond the 5′-end of downstream DNA. Entry into the duplex DNA region will lead to the formation of a 5′-flap. As the 5′- and 3′-ends of the nascent DNA are not properly aligned, the ligase cannot catalyse the formation of a new phosphodiester bond and seal the strand break. In agreement with these *in vitro* findings, exonuclease-deficient mutator mice contain high levels of nicked mtDNA molecules. As predicted, these nicks are enriched in the H-strand near OriH, the site where H-strand DNA synthesis is both initiated and terminated.

Failure to ligate the H-strand close to OriH will cause problems during the next round of replication. A nick at OriH will not impair initiation of H-strand DNA synthesis, as the nick is in the non-template strand. However, once the nascent L-strand initiated at OriL reaches the nicked H-strand DNA at OriH, a double-strand break will be formed. The break will cause the formation of linear mtDNA fragments spanning OriH and OriL, as identified in mutator mice and MGME1-deficient patients (Fig. 5d).

In contrast to the strand-specific nicks observed in the OriH region, a similar phenomenon is not observed at OriL. This difference is explained by the strand displacement mode of mtDNA replication, which states that H-strand DNA synthesis precedes and is required for OriL activation. When DNA synthesis initiated at OriL reaches a nick in the H-strand, a double-stranded break is formed, causing the linear fragment. Therefore, L-strand DNA synthesis events initiated on a template with a nick in the H-strand will not go full circle, which is required for nick formation near OriL. Furthermore, in the more unlikely event that a molecule with a nick in the L-strand near OriL is formed, it cannot be used as a template for subsequent rounds of mtDNA replication. On this nicked template, DNA synthesis initiated at OriH will generate a double-stranded break at OriL. DNA synthesis will be terminated and no mtDNA daughter molecules will be formed (Supplementary Fig. 7).

We here characterize disease-causing POLγA mutations that impair exonuclease activity^35^. In our work, we characterized seven mutations and found that three of these (G303R, L304R and S305R) abolished exonuclease activity and impaired ligation as a result of unchecked strand-displacement activity. The mutations map downstream of the exonuclease motif II in the amino-terminal region of POLγA, which forms part of a highly conserved DNA-binding channel^36^. Interestingly, we also identified one mutant (R232H) with markedly increased exonuclease activity, which resulted in the opposite molecular phenotypes, that is, reduced strand-displacement activity and more efficient formation of ligatable ends.

EXO- POLγ does not have a dominant-negative function and ligation is still possible in the presence of WT POLγ *in vitro* (Supplementary Fig. 6). Furthermore, this observation may also explain why patients with these mutations lack linear deletions, since nearly all patients with mutations in the exonuclease domain are heterozygous, the only exception being patients homozygous for the L304R mutation^37^. Compared with the EXO- mutation, L304R has relatively low polymerase activity and very limited strand-displacement activity (Figs 3d and 4b). The reduced processivity may prevent L304R from performing excessive strand displacement and instead stall within a few nucleotides of entering duplex DNA. In effect, the reduced POLγ processivity therefore prevents the generation of long, non-ligatable DNA flaps. The reduced POLγ processivity also helps to explain the depletion of mtDNA observed in these patients^37^.

Exonuclease activities are also important for the formation of ligatable nicks in other systems. For instance, Pol δ, the lagging-strand nuclear DNA polymerase, displaces two to three nucleotides of a downstream duplex DNA, before it backs up to the nick using its 3′–5′ exonuclease activity^14^. The two steps of polymerization and excision are reiterated in a process called idling, which is essential for efficient ligation. In our strand-displacement assays, we found that WT POLγ behaves in a similar way. The polymerase enters several nucleotides into the duplex downstream DNA region and then backs up in an exonuclease-dependent manner towards the nick. The G303R, L304R and S305R mutations are impaired in their ability to back up to the nick, thus leaving a 5′-flap that is incompatible with ligation. The fact that the formation of DNA flaps is deleterious is also reflected by the presence of several different flap nucleases in cells. FEN1, DNA2, EXOG and MGME1 are all nucleases with mitochondrial localization that have all been shown to remove 5′-flaps^18^,^38^,^39^,^40^,^41^,^42^,^43^,^44^. Flap nucleases may partially rescue the displacement activity of exonuclease-deficient POLγ mutants, as their flap removal activity would allow for subsequent ligation. Mutations in flap endonucleases may themselves impair ligation. As mentioned above, linear fragments similar to those seen in the mutator mouse have also been identified in MGME1 patient fibroblasts^34^. MGME1 has been implicated in primer removal at OriH, a process that must be completed before ligation. Failure to remove the primer will cause ligation problems and nicks in the H-strand at OriH, thus possibly explaining why MGME1 patients have the same linear fragments as those seen in mutator mice. One may speculate that the ligation defects observed here contribute to the premature ageing phenotype observed in mutator mice. For instance, creation of ligatable ends is a prerequisite for functional base-excision repair. To address this question, it is necessary to identify POLγ mutations that increase the mtDNA mutation load without affecting mtDNA ligation. In future work, we will try to identify such mutations and investigate their effects on mammalian ageing.

## Methods

### Recombinant proteins

Recombinant baculoviruses encoding TWINKLE, mitochondrial Lig3, POLγB and the different POLγA versions were expressed in Sf9 cells^21^. These recombinant proteins all lacked the N-terminal mitochondrial targeting sequence and carried a carboxy-terminal 6 × His-tag. The proteins were purified over HIS-Select Nickel Affinity Gel (Sigma-Aldrich) and HiTrap Heparin HP (GE Healthcare), followed by HiTrap SP HP or HiTrap Q HP columns (GE Healthcare), depending on the net electrical charge of the protein. MtSSB lacking the N-terminal mitochondrial targeting sequence was expressed in insect cells and purified over DEAE Sepharose Fast Flow (GE Healthcare), HiTrap Heparin HP and HiTrap SP HP, followed by gel filtration using HiLoad Superdex 200 (GE Healthcare).

### DNA substrates

For assays using linear substrates (Figs 1b, 2d, and 4a–c, [Fig f4]), an 80-bp-long gapped substrate was created by annealing three oligonucleotides together: an 80-nt template strand (5′-GTT ATC TAA GCT GCT CTT GGT AGG CAT TGA CGT CCA TAC TGC AAA TTC AGC TCT GTG CAG TTA GGC AGG AGT CTA CAA GC-3′) was annealed to a 30-nt upstream priming oligonucleotide (5′-GCT TGT AGA CTC CTG CCT AAC TGC ACA GAG-3′) and a 30-nt downstream blocking oligonucleotide (5′-TCA ATG CCT ACC AAG AGC AGC TTA GAT AAC-3′), resulting in the formation of a 20-nt gap. Oligonucleotides were labelled as indicated, using T4 polynucleotide kinase and [γ-^32^P] ATP.

### Strand-displacement assay

Strand-displacement reactions (Figs 1b and 4a,b) were performed on the 80-bp gapped substrate with the upstream primer labelled on the 5′-end. Reactions (20 μl) contained 25 mM Tris-HCl, 10 mM MgCl_2_, 0.1 mg ml^−1^ BSA, 1 mM dithiothreitol (DTT), 0.5 mM ATP, 100 μM each of dNTPs, 25 fmol DNA substrate, 200 fmol mtSSB, 600 fmol POLγB and 150 fmol POLγA. Reactions were incubated for the indicated times at 32 °C and analysed by electrophoresis in 7 M urea/10% polyacrylamide gels. For quantification of complete strand displacement (Supplementary Fig. 2) the same substrate was used, but the downstream blocking oligonucleotide was labelled instead of the upstream primer. Reactions were incubated for the times indicated at 32 °C and analysed by electrophoresis in 10% native polyacrylamide gel and signals were visualized by autoradiography. Gelquant software (Bio-Imaging Systems) was used for quantification of band intensities where the completely displaced band was compared with the substrate band. *P*-values were calculated with the unpaired Student's *t*-test or one-way analysis of variance using the SigmaPlot software. Statistical analyses were based on three or more independent experiments.

### Coupled gap filling and ligation assay

Coupled gap filling–ligation assays (Figs 2d and 4c) were performed as the strand-displacement assay, but with the downstream blocking oligonucleotide labelled. Where indicated, 1 unit of T4 DNA ligase or 25 fmol DNA ligase 3 were added to the reactions. Reactions were analysed by 7 M urea/10% polyacrylamide gels and visualized by autoradiography. The intensities of the ligated bands were compared with the substrate band, where the latter was based on reactions where no ligase was added.

### Coupled second-strand synthesis and ligation assay

A ^32^P-labelled 32 nt oligonucleotide (5′-CTA TCT CAG CGA TCT GTC TAT TTC GTT CAT CC-3′) was annealed to single-stranded pBluescript SK (+) (Figs 1b and 3d). DNA synthesis assays were performed using 10 fmol template, 150 fmol of indicated POLγA version, 600 fmol POLγB and 10 pmol mtSSB in 25 mM Tris–HCl (pH 7.8), 1 mM DTT, 10 mM MgCl_2_, 0.1 mg ml^−1^ BSA, 100 μM of each dNTP. One unit of T4 DNA ligase was added when indicated. Reactions were incubated at 37 °C for the indicated times and stopped by the addition of 4 μl of stop buffer (90 mM EDTA, 6% SDS, 30% glycerol, 0.25% bromophenol blue and 0.25% xylene cyanol). Samples were separated on a 0.9% agarose gel containing 0.5 μg ml^−1^ EtBr and visualized by autoradiography.

### 3′–5′ Exonuclease activity assays

To measure exonuclease activity, we annealed a 32-base oligonucleotide (5′-CTA TCT CAG CGA TCT GTC TAT TTC GTT CAT CG-3′) ^32^P-labelled at the 5′-end to single-stranded pBluescript SK+, creating a 31-bp dsDNA region with a one-nucleotide mismatch at the 3′-end (Fig. 3c). The reactions were performed as described previously^45^, but with 150 fmol of the indicated POLγA version and 600 fmol of POLγB. The products were analysed by electrophoresis in 7 M urea/20% polyacrylamide gels and visualized by autoradiography.

### EMSA and coupled 3′–5′ exonuclease/polymerase assays

DNA-binding affinity (Fig. 3b) of POLγ to a primed DNA template was assayed using an electrophoretic mobility shift assay (EMSA)^22^. A 20-base oligonucleotide (5′-CGG TCG AGT CTA GAG GAG CC-3′) was labelled at the 5′-end with [γ-^32^P] ATP and annealed to a 35-base complementary oligonucleotide (5′-GAC TAC GTC TAT CCG GGC TCC TCT AGA CTC GAC CG-3′), to produce a primed DNA template with a 15-base single-stranded 5′-tail. Reactions were carried out in 15 μl volumes containing 10 fmol DNA template, 20 mM Tris–HCl pH 7.5, 1 mM DTT, 0.1 mg ml^−1^ BSA, 10 mM MgCl_2_, 10% glycerol, 3 mM dCTP, 0.3 mM ddGTP, 450 fmol of the indicated version of POLγA and 1.8 pmol POLγB, where indicated. Proteins were added as indicated in the figure legends and reactions were incubated at room temperature for 10 min before separation by electrophoresis in 6% non-denaturing polyacrylamide gels in 0.5 × TBE buffer for 1 h at 100 V.

The EMSA substrate was also used to examine the polymerization and 3′–5′ exonuclease activities of POLγA (Supplementary Fig. 4). Coupled 3′–5′ exonuclease/polymerase reactions contained 10 fmol of the DNA template, 20 mM Tris–HCl pH 7.5, 10% glycerol, 1 mM DTT, 10 mM MgCl_2_, 100 μg ml^−1^ BSA, 150 fmol of POLγA and 600 fmol of POLγB, and the indicated concentrations of the four dNTPs. Reactions were incubated at 37 °C for 15 min and stopped by the addition of gel-loading buffer. Samples were analysed on a 15% denaturing polyacrylamide gel in 1 × TBE buffer for 2 h at 160 V.

### DNA isolation and Southern blot analyses

Genomic DNA was isolated from MEF by addition of lysis buffer (10 mM Tris–HCl, pH 8, 100 mM NaCl, 25 mM EDTA, 0.5% SDS) and proteinase K to cell pellets. After incubation for 1 h at 42 °C, total DNA was extracted by phenol/chloroform extraction and ethanol precipitation. When indicated, total DNA (1–3 μg) was cut with XhoI to linearize mtDNA or with Nt.BbvCI to produce nicked mtDNA circles. The DNA was separated on 0.4% or 0.5% agarose gels in 1 × TBE buffer in the presence or the absence of 0.5 μg ml^−1^ EtBr, followed by transfer onto a nylon Hybond-N+ membrane (GE Healthcare). PCR fragments of mtDNA located in the minor arc (mtDNA position 3,000–3,700) or major arc (mtDNA position 8,461–9,301) were used as probes to detect mtDNA and signals were visualized by autoradiography. Radioactive labelling of Southern probes was done according to the manufacturer's instructions (Prime-IT II Random Prime Labeling Kit, Agilent). Large orifice tips were used to diminish unwanted nicking of the DNA during preparation.

### Strand-specific ddPCR

Strand-specific ddPCR was used to quantify individual DNA strands in mtDNA. Three primers were used in each reaction: Tag, Tagging and Reverse as listed in Supplementary Table 1. Primers with ‘H' and ‘L' were used for amplification of the heavy- and light-strand DNA sequences, respectively. The reaction was initiated by annealing of the Tagging primer, which contains 11–13 nt of locus-specific sequence at the 3′-end, and a 19 nt Tag sequence at the 5′-end. The annealed Tagging primer was first extended after which the generated hybrid sequence was amplified by the other two primers (Tag and Reverse) at proper temperatures. The reactions contained 400 nM of Tag and Reverse primers, and 8 nM Tagging primer in a 25-μl reaction volume. After an initial cycle of 95 °C for 3 min, 40 °C for 5 min, increase to 72 °C at 2 °C min^−1^, 94 °C for 4 min, we continued with an additional 40 cycles of 94 °C for 15 s and 67 °C for 1 min. The PCR reactions were performed with ddPCR (Bio-Rad, QX200). Genomic DNA extracted from WT and mutator mouse liver was digested by either EcoRI (no cutting site in the amplified regions) or XbaI (with one cutting site in the amplified region). All animal work was performed in strict accordance with the recommendations and guidelines of the Federation of European Laboratory Animal Science Associations (FELASA). The permit was approved by the ‘Landesamt für Natur, Umwelt und Verbraucherschutz Nordrhein-Westfalen', Germany. ddPCR results from XbaI-digested genomic DNA was used as background. The H-to-L strand ratio in mtDNA from mutator mice was calculated and normalized to the values obtained from WT controls. The experiments were repeated three times. *P*-values were calculated in Excel (Microsoft). The primer sequences are listed in Supplementary Table 1.

## Additional information

**How to cite this article:** Macao, B. *et al.* The exonuclease activity of DNA polymerase γ is required for ligation during mitochondrial DNA replication. *Nat. Commun.* 6:7303 doi: 10.1038/ncomms8303 (2015).

## Supplementary Material

Supplementary Information Supplementary Figures 1-7, Supplementary Table 1
