# Supplementary material for: The exonuclease activity of DNA polymerase γ is required for ligation during mitochondrial DNA replication
Source: Nat Commun. 2015 Jun 22;6:7303. doi: 10.1038/ncomms8303 (PMC4557304; doi:10.1038/ncomms8303)
Supplement: Supplementary Information — Supplementary Figures 1-7, Supplementary Table 1 [file ncomms8303-s1.pdf]

Supplementary Figure 1

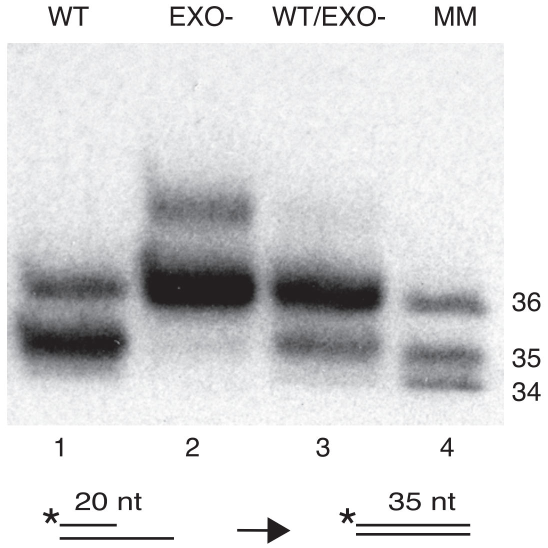

Supplementary Figure 1. EXO- POLγ has increased terminal transferase activity

DNA synthesis was initiated from a 20 nt primer, which had been radioactively labeled in the 5′-end and annealed to a 35 nt oligonucleotide template (lower panel). The majority of WT POLγ terminated at 35 nt, but a weaker 36 nt band was also produced (lane 1). EXO- POLγ mainly terminated at 36 nt, adding one extra terminal nucleotide, and the 35 nt product was barely detectable (lane 2). Lane 3 contains both WT and EXO- POLγ (1:1 ratio). Samples were separated on a 12.5 % denaturing PAGE.

Supplementary Figure 2

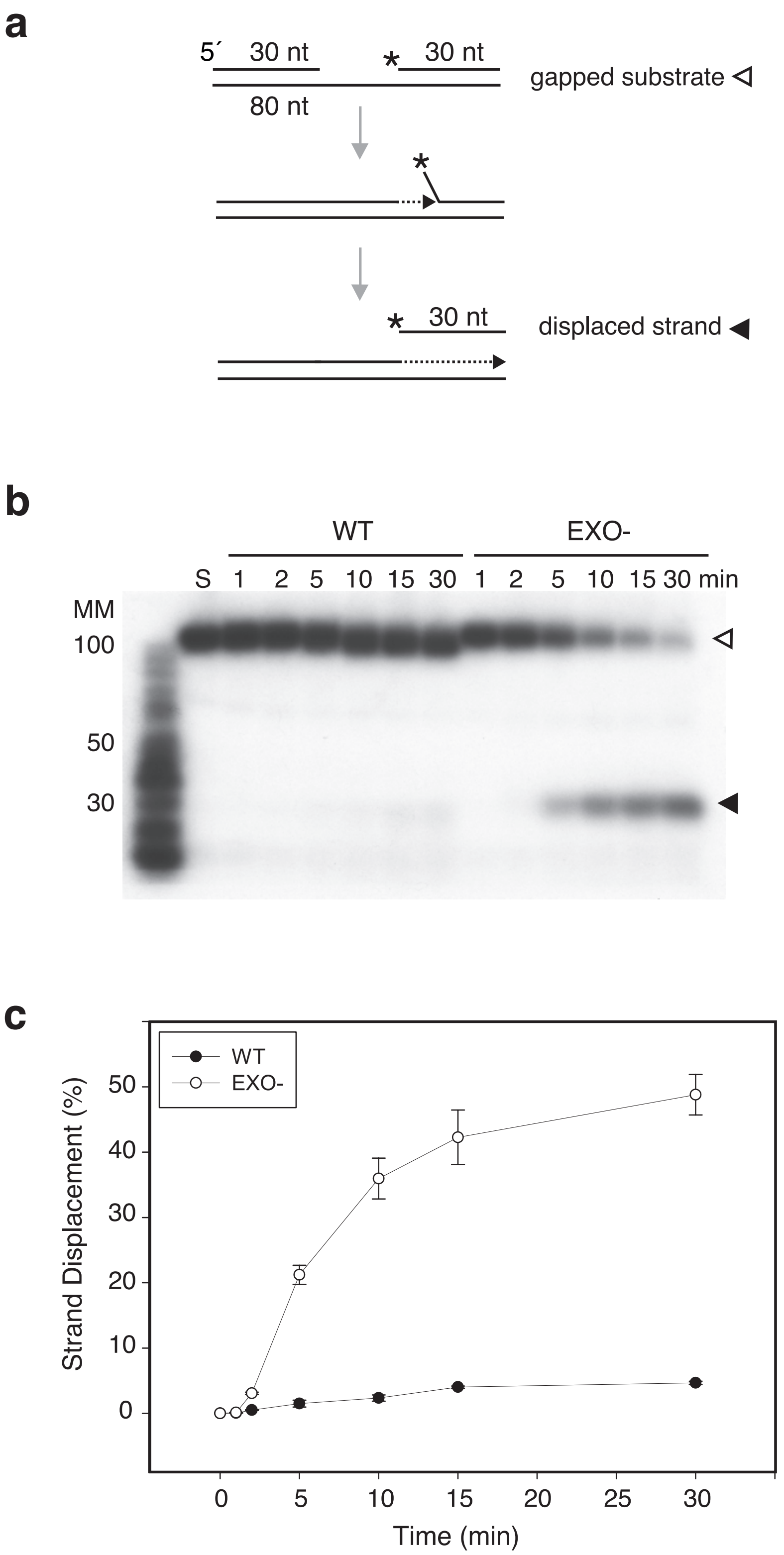

Supplementary Figure 2. EXO- POLγ displays increased strand displacement activity
(a) Diagram of the linear gapped substrate used in strand displacement assays, with the progression of displacement illustrated below. (b) EXO- POLγ displaces the downstream 30 nt oligonucleotide (black arrow) more readily than WT POLγ. (c) Quantification of strand displacement by WT and EXO- POLγ measured as the intensity of the upper band, corresponding to the displaced strand (black arrowhead) relative the lower band, corresponding to the substrate (white arrowhead). Lane S contains the substrate with no POLγ added. Values are mean ± SEM.

Supplementary Figure 3

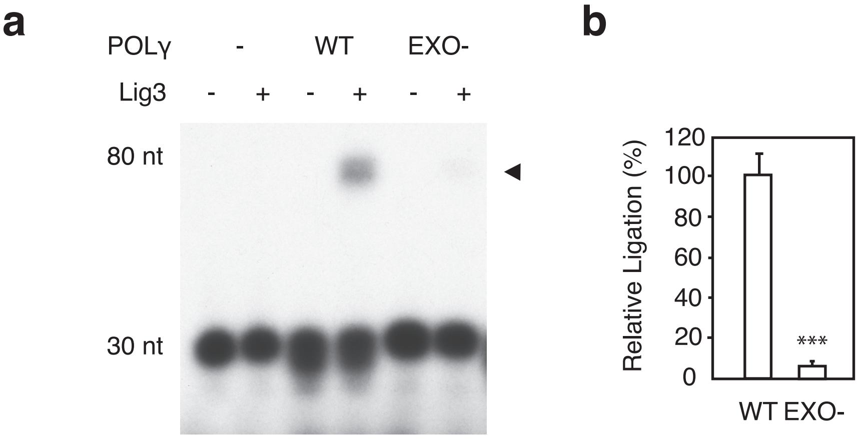

Supplementary Figure 3. The POLγ exonuclease activity is required for efficient ligation by mitochondrial Lig3

(a) A coupled replication-ligation assay in the presence of Lig3 performed as described in Fig. 2 c and d. Lower levels of the 80 nt long, ligated product (indicated with arrowhead) is formed when EXO- POLγ is used instead of WT POLγ. (b) Quantification of ligation efficiency in panel a. The levels of the 80 nt, ligated product was normalized to the substrate band incubated with the corresponding POLγ, but in the absence of Lig3. Mean values ± SEM, $P \leq 1.5 \times 10^{-5}$ (Student´s unpaired t-test).

Supplementary Figure 4

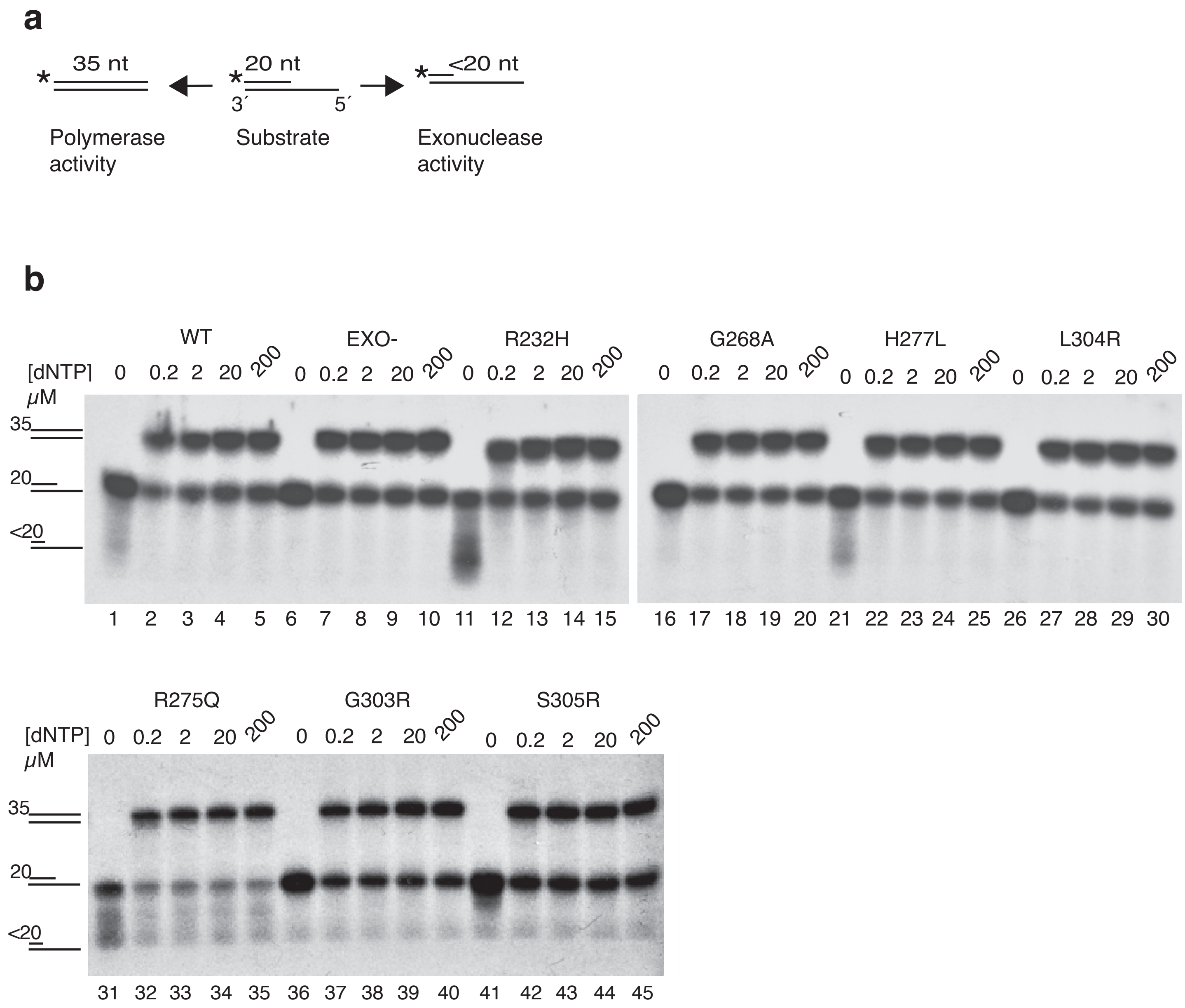

Supplementary Figure 4. Polymerase and exonuclease activities of the POLγ mutant proteins
The polymerase and exonuclease activities of the different POLγA mutant proteins were monitored using a short, primed oligonucleotide substrate (a) in the presence of increasing amounts of dNTPs (0 - 200 μM). (b) In the absence of dNTPs, EXO-, G268A, G303R, L304R, and S305R mutants showed very low exonuclease activity. The R232H mutant had increased exonuclease activity (lane 11). With increasing amounts of dNTPs all polymerases were able to extend the primer as efficiently as WT.

Supplementary Figure 5

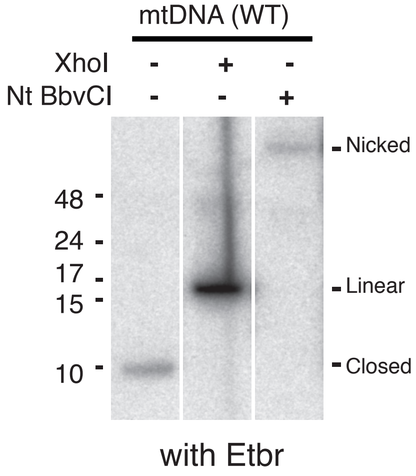

Supplementary Figure 5. Verification of migration pattern of mouse mtDNA
Genomic DNA was linearized with XhoI or nicked with Nt.BbvCI. The samples were analyzed on a 0.4 % agarose gel in the presence of ethidium bromide. The presence of ethidium bromide in the agarose gel facilitates differentiation between nicked, linearized and closed circular dsDNA molecules. The mtDNA was detected by southern blotting using a probe against the minor arc. Lambda DNA, monoCut mix was used as a molecular marker.

Supplementary Figure 6

a

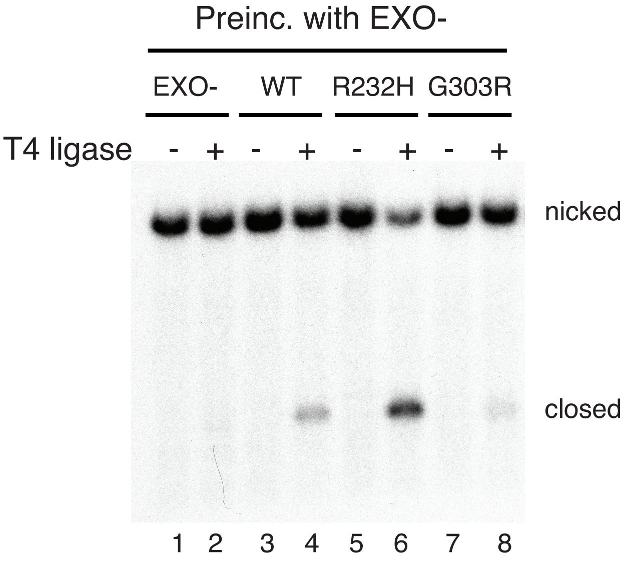

b

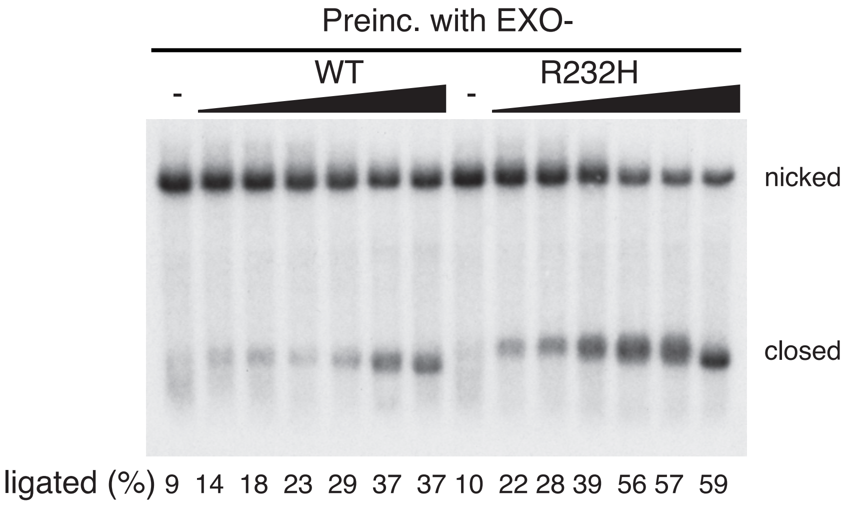

Supplementary Figure 6. POLγ with functional exonuclease activity can rescue exonuclease deficent POLγ in trans

(a) A primed circular ssDNA was preincubated with EXO- POLγ (lanes 1-8) to produce circular dsDNA with a non-ligatable nick. After 5 minutes incubation indicated POLγ version was added and the reaction was allowed to proceed for additional 10 min. T4 DNA ligase was added when indicated. (b) The R232H mutant is more effective than WT POLγ in rescuing the ligation defect of EXO- POLγ. The reactions were preincubated with EXO- POLγ (150 fmol) as in (a) and increasing amount of WT or R232H protein (0 fmol, 37,5 fmol, 75 fmol, 150 fmol, 300 fmol, 600 fmol or 1200 fmol) was added together with T4 DNA ligase. The ligation efficiency is indicated below each lane and corresponds to the percentage of the ligated product (closed) relative the unligated substrate (nicked).

Supplementary Figure 7

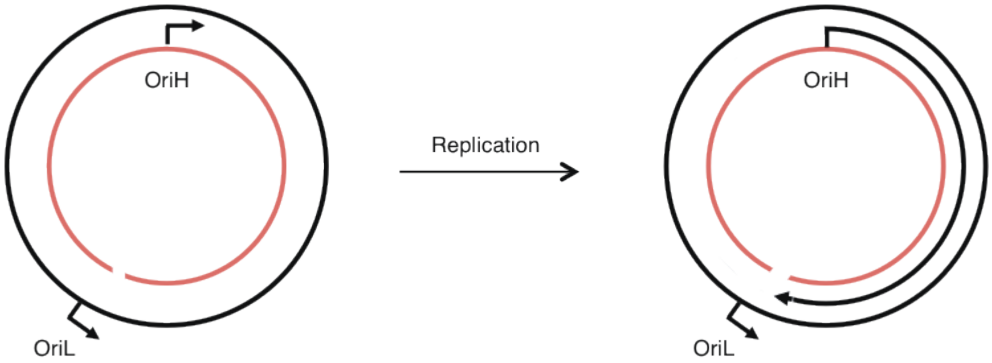

H-strand DNA synthesis is terminated already at OriL and no full circle DNA products are formed

Supplementary Fig. 7. MtDNA molecules with a nicked L-strand cannot be propagated
H-strand mtDNA synthesis is initiated at OriH. Once the nascent H-strand reaches the nick, a double stranded break is formed and mtDNA synthesis is terminated before OriL activation. No new daugther molecules are formed. The parental L-strand is indicated in red.

Supplementary Table 1

Primer sequences used in ddPCR

Gene name/region	Primer Name	Sequence
OriL	Tag-H-OL	AAG GAG CGC AGC GCC TGT ACT G
	Tagging-H-OL	AAG GAG CGC AGC GCC TGT ACT GGC TTC AAT CT
	Reverse-H-OL	CAG GCT CCG AAT AGT AGA TAG AGG GTT CCG A
	Tag-L-OL	AAG GAG CGC AGC GCC TGT AGA T
	Tagging-L-OL	AAG GAG CGC AGC GCC TGT AGA TAG AGG GTT CC
	Reverse-L-OL	ACC CTA TTA CTG GCT TCA ATC TAC TTC TAC CGC
16S	Tag-H-16S	AAG GAG CGC AGC GCC TGT AAC G
	Tagging-H-16S	AAG GAG CGC AGC GCC TGT AAC GGC TAA ACG A
	Reverse-H-16S	TCT CCG AGG TCA CCC CAA CCG A
	Tag-L-16S	AAG GAG CGC AGC GCC TGT ATC T
	Tagging-L-16S	AAG GAG CGC AGC GCC TGT ATC TCC GAG GTC A
	Reverse-L-16S	CGG CTA AAC GAG GGT CCA ACT GTC TCT
OriH	Tag-H-OH-1	AAG GAG CGC AGC GCC TGT ACA G
	Tagging-H-OH-1	AAG GAG CGC AGC GCC TGT ACA GCA CAC AGT C
	Tag-HOH-2	AAG GAG CGC AGC GCC TGT AGAT
	Tagging-H-OH-2	AAG GAG CGC AGC GCC TGT AGAT GGT ATC GGG T
	Reverse-H-OH-1 &2	TTA AGA GGA GGG GGT GGG GGG TTT
	Tag-L-OH-1&2	AAG GAG CGC AGC GCC TGT AAA G
	Tagging-L-OH-1&2	AAG GAG CGC AGC GCC TGT AAAG AGG AGG GGG T
	Reverse-L-OH-2	GAA GAA GGA GCT ACT CCC CAC CAC CA
	Reverse-L-OH-1	TGA AAG GAC AGC ACA CAG TCT AGA CGC
COII	Tag-H-COII	AAG GAG CGC AGC GCC TGT AAT A
	Tagging-H-COII	AAG GAG CGC AGC GCC TGT AAT AAC CGA GTC G
	Reverse-H-COII	GTT GCT TGA TTT AGT CGG CCT GGG A
	Tag-L-COII	AAG GAG CGC AGC GCC TGT ATT A
	Tagging-L-COII	AAG GAG CGC AGC GCC TGT ATT AGT CGG CCT
	Reverse-L-COII	GAA GTT GAT AAC CGA GTC GTT CTG CCA ATA GA
ND5	Tag-H-ND5	AAG GAG CGC AGC GCC TGT AAT A
	Tagging-H-ND5	AAG GAG CGC AGC GCC TGT AAT ACT TTG CCT C
	Reverse-H-ND5	TGT CTT GTT CGT CTG CCA GGC TAT GA
	Tag-L-ND5	AAG GAG CGC AGC GCC TGT ATT T
	Tagging-L-ND5	AAG GAG CGC AGC GCC TGT ATT TCG GAT GTC TT
	Reverse-L-ND5	TGC CTC GGA GCC CTA ACC ACA TTA TT
